# Chain mediations of perceived social support and emotional regulation efficacy between role stress and compassion fatigue: insights from the COVID-19 pandemic

**DOI:** 10.3389/fpubh.2023.1269594

**Published:** 2023-11-13

**Authors:** Yuan Zhang, Huijuan He, Chongming Yang, Xiangrong Wang, Jiang’an Luo, Jie Xiao, Bei Fu, Yiwen Chen, Chenjuan Ma

**Affiliations:** ^1^School of Nursing, Hubei University of Chinese Medicine, Wuhan, Hubei, China; ^2^College of Family, Home, and Social Sciences, Brigham Young University, Provo, UT, United States; ^3^NYU Rory Meyers College of Nursing, New York, NY, United States

**Keywords:** COVID-19, compassion fatigue, emotional regulation efficacy, nurses, perceived social support, role stress

## Abstract

**Background:**

Nurses at the frontline faced high risks of the COVID-19 infection, undertook heavy workloads of patient care, and experienced tremendous stress that often led to compassion fatigue.

**Aim:**

This study was to explore the role of positive psychosocial resources (i.e., perceived social support and emotional regulation efficacy) in the relationship between role stress and compassion fatigue.

**Methods:**

A cross-sectional design was conducted in Hubei Province, China between May and September 2021. The Role Stress Questionnaire, the Perceived Social Support Scale, the Emotional Regulation Efficacy Scale, and the Professional Quality of Life Scale were used to measure key variables of interest. Nurse socio-demographic data were also collected. Structural equation modeling was used to explore the relationships, including potential mediating effect, among role stress, perceived social support, emotional regulation efficacy, and compassion fatigue.

**Results:**

A total of 542 nurses participated in this investigation, and 500 were eventually enrolled in the analysis. The incidence of compassion fatigue among nurses was 94.2%, including 65.8% of nurses reporting at least moderate compassion fatigue. Univariate analysis showed that educational level, marital status, hospital rank, sleep time were the factors affecting compassion fatigue of the nurses. The structural equation modeling revealed that: Role stress had a direct positive effect on compassion fatigue; Perceived social support and emotional regulation efficacy partially mediated the link between role stress and compassion fatigue respectively; And there was a chain mediating role of perceived social support and emotional regulation efficacy between role stress and compassion fatigue.

**Conclusion:**

The incidence of compassion fatigue was high during the COVID-19 pandemic among bedside nurses in China. Improving social support and enhancing the efficacy of emotion regulation may help alleviate compassion fatigue directly and/or via buffering the impact of role stress.

## 1. Introduction

The sudden outbreak of the COVID-19 pandemic was an unexpected public health crisis and posed dramatic challenges to healthcare system around the world (1). Nurses were called to fight against the COVID-19 pandemic at the frontline. In this silent yet brutal battle, nurses experienced fears of infections, unexpected changes in the type of work, exhaustion due to heavy workloads, feelings of inadequate support, and uncertainty about disease control (2–4). Furthermore, they also experienced unprecedented psychological impact from the deaths of patients they cared for (5). Both the physical and mental challenges during the COVID-19 pandemic expose bedside nurses to an extremely high risk of suffering from compassion fatigue.

“Compassion fatigue” was first introduced in 1992 to describe nurses’ burnout and it has received attention from the healthcare industry since then (6). According to Figley (7), compassion fatigue is defined as the secondary trauma experienced by helpers when providing assistance to recipients with empathy as a premise, which leads to a decreased interest and ability in empathizing with oneself, as well as a sense of fatigue from the work of helping. This sense of fatigue can not only alter the original work values of helpers, but also is accompanied by a range of psychological discomfort symptoms. Compassion fatigue can result in poor health outcomes of nurses, such as insomnia, reduced immunity, headaches, sleep disorders, etc. It can also impact nurses’ performance at work, such as reduced quality of care, missed care, and medical errors (8–10). During the COVID-19 pandemic, research has focused primarily on investigating the current state of compassion fatigue (i.e., prevalence, associated factors) and the need to improve compassion fatigue in healthcare workers (i.e., neutralizing emotional trauma, utilizing relaxation strategies, improving capacity to respond to disasters and teaching coping strategies for dealing with psychological problems) (11, 12). However, few studies have explored the mechanisms by which compassion fatigue occurs during the COVID-19 pandemic. In addition, as an important healthcare workforce, nurses bear a heavy burden in clinical care and public prevention (13). Therefore, it is necessary to explore the mechanism of compassion fatigue among nurses to promote their physical and mental health.

To date, compassion fatigue has been linked to work-related stress as a main risk factor for developing compassion fatigue. One common source of work-related stress is the roles that arise from perceived multiple expectations within a social structure (14). When the expectations are conflicting, ambiguous, or overloaded, individuals experience role stress (14). Due to long working hours, heavy workload, fear of infecting their relatives and concerns about seeing patients die, nurses working during the pandemic experienced high role stress (3, 4, 15, 16). Indeed, studies have demonstrated that role stress has a strong connection with compassion fatigue (8, 17). In a study of Spanish medical staff, Ruiz-Fernández (18) and his team found that higher role stress was likely accompanied with more compassion fatigue. However, the strength of such relationship is unclear among nurses during the COVID-19 pandemic.

Recently, a series of studies have been conducted to identify potentially protective factors of compassion fatigue. Among many explored factors, psychosocial resources have drawn attention to the research community regarding their roles in compassion fatigue development. Psychosocial resources represent individual and relational factors with intrinsic values to promote physical and mental health (19). Positive psychosocial resources can help individuals recover quickly after experiencing negative events, especially major trauma, difficulties, setbacks, difficulties, stress, and even life-threatening situations (13). Psychosocial resources can effectively alleviate the effect of physical and psychological distress on compassion fatigue (13, 20, 21).

Perceived social support is one type of psychosocial resources. It is an essential component of a person’s social support system and can be defined as an emotional experience of individuals who believe they are respected, supported, and understood in society and receive support from others (22). Perceived social support is widely referred as a protective factor for psychosocial adjustment. It is believed that the establishment of social support platforms may effectively buffer the impact of the COVID-19 pandemic on the mental health of healthcare workers (3, 22). For instance, Polish nurses who had higher levels of perceived social support tended to have lower levels of compassion fatigue (23). Social support also helps individuals better manage consequent emotional damage in stressful situations (23–25).

Another psychosocial resource is emotional regulation efficacy, which can be defined as an individual’s confidence in his or her competence to regulate emotions. It can reduce emotional tension, relieve anxiety and depression, and promote mental health (26). The ability to effectively regulate emotions may alleviate the potential adverse effects when people encounter difficulties in public health emergencies (27). Appropriate emotional regulation abilities were found to reduce compassion fatigue in Iranian nurses in the COVID-19 outbreak (28). Adaptive emotion regulation strategies can buffer negative feelings caused by stress and enhance personal well-being (29). Similarly, individuals who were more able to manage their emotions perceived lower levels of stress which is a critical risk factor of compassion fatigue (30). Thus, the two psychosocial resources, perceived social support and emotional regulation efficacy, appeared to mediate the relationship between nurse role stress and compassion fatigue. However, it is unclear to what extent such complex relationships hold among Chinese nurses in public health emergencies particularly like the COVID-19 pandemic.

**Figure 1:**
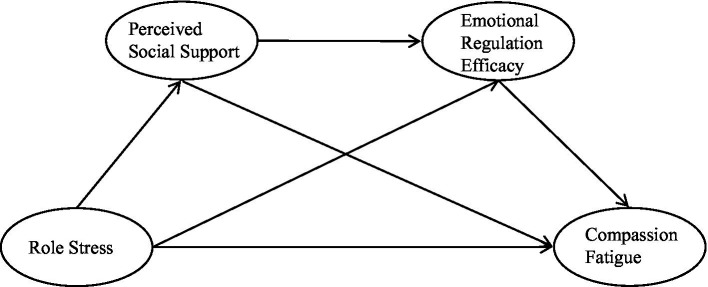
Hypothesized mediation model.

This study was aimed to examine the relationships between role stress, two psychosocial resources factors (perceived social support and emotional regulation efficacy), and compassion fatigue during the COVID-19 pandemic. We hypothesized that role stress was positively and directly associated with compassion fatigue (H1); perceived social support mediated the relationship between role stress and compassion fatigue (H2); The relationship between role stress and compassion fatigue was mediated by emotional regulation efficacy (H3); and perceived social support and emotional regulation efficacy exert chain-mediating roles in the influence of role stress on compassion fatigue (H4). These hypotheses are depicted in Figure 1.

## 2. Materials and methods

This study was carried out with adherence to the STROBE guidelines after it had been approved by the institutional review board (Approval: HBZY2020-C14-01) and in accordance with the Declaration of Helsinki (31).

### 2.1. Study design

This study used a cross-sectional design.

### 2.2. Study population

The study population was registered nurses recruited from seven hospitals in three cities (Wuhan, Jingzhou, and Hanchuan) in Hubei Province. The participants were selected for the study if they had provided direct patient care during the pandemic and from May to September, 2021. Advanced practice nurses and intern nurses were excluded from this study.

### 2.3. Data collection

Data for this study were collected online anonymously. To ensure the completeness of survey responses, the participants had to answer each question before they could submit the survey. Upon receiving the completed questionnaires, two researchers checked the quality of the data. The questionnaires were excluded if they included identical responses to the majority of the questions or response times were unreasonably short.

### 2.4. Sample size

A power analysis was conducted to estimate the minimum sample size required in this study using the following sampling formula: $n=\frac{{\left(Z_{\frac{\alpha }{2}}\right)}^{2}p\left(1-p\right)}{d^{2}}$ (32), where *α* = 0.05, Z = confidence level ($\frac{\alpha }{2}$), p = prevalence, and d = marginal error. The incidence of compassion fatigue was estimated at 50% according to a cross-sectional study by Kabunga et al. (33). With a 95% confidence level, a margin of error of 0.05, and considering a 20% sample attrition rate, it was estimated that at least 461 questionnaires should be distributed to participants.

### 2.5. Measures and survey instruments

#### 2.5.1. Role stress questionnaire

The Role Stress Questionnaire, which was developed by Peterson et al. (34) and then translated and revised it into Chinese by Li et al. (35), was used to measure the level of role stress nurse participants experienced. This questionnaire consists of 13 items (e.g., “I often face situations where my goals conflict with each other”). It contains three sections: role conflict (3 items), role ambiguity (5 items), and role overload (5 items). Each item is scored on a 5-point Likert scale from 1 (strongly disagree) to 5 (strongly agree), with the five items of role ambiguity being scored reversely. To determine the level of role stress, a total score was calculated, with a higher score indicating a higher level of role stress. The Cronbach alpha of this scale 0.838 suggested a high reliability in this study.

#### 2.5.2. Perceived social support scale

Nurses’ perceived social support was measured using the Chinese version of the Perceived Social Support Scale. Zimet et al. (36) developed the original version of the Perceived Social Support Scale, which has been translated into Chinese by Jiang (37). The scale includes 12 items (e.g., “I can rely on my friends in times of difficulty”) in three dimensions: family support (4 items), friend support (4 items), and other support (4 items). Participants rated how much support they had received from family, friends, and others on a 7-point Likert scale ranging from 1 (strongly disagree) to 7 (strongly agree). A total score of the scale reflects the overall level of support. As the score increased, the perception of social support improved. The Cronbach alpha (0.972) indicated a high reliability in this study.

#### 2.5.3. Emotional regulation efficacy scale

The Emotional Regulation Efficacy Scale by Caprara et al. (38) was developed to assess nurses reported emotion regulation efficacy. Chinese researchers adapted this scale to the needs of the Chinese population for measuring individuals’ efficacy of emotion regulation (39). The scale consists of 17 items (e.g., “I express my pleasure when something pleasant happens”) in total and includes two subscales, the efficacy to express positive emotions (POS) and the efficacy to manage negative emotions (NEG). POS is composed of 6 questions for two sub-dimensions: efficacy to convey happy/excited emotions and efficacy to express proud emotions. The NEG consists of 11 questions, including managing anger/rage, frustration/pain, and guilt/shame. The scale is scored on a 5-point Likert scale ranging from 1 (very unlikely) to 5 (very likely). The scores were calculated for each subscale (i.e., POS and NEG) as the sum of individual items in each of them. Higher scores reflected greater efficacy of emotion regulation. The Cronbach alpha of the two subscales (0.934 & 0.951) indicated high reliability of emotion regulation efficacy.

#### 2.5.4. Chinese version of professional life quality scale (ProQOL)

Compassion fatigue among participating nurses was measured using the Chinese Version of the Professional Life Quality Scale (ProQOL). The original ProQOL was developed by Figley (7) and was later adapted to Chinese by Chen et al. (40). The Chinese version has been used to measure compassion fatigue status among doctors, nurses, chaplains, police officers, and rescue workers. This 30-item scale (e.g., “I am Satisfied with my job”) contains three subscales: compassion satisfaction (10 items), burnout (10 items), and secondary trauma (10 items). Items 1, 4, 15, 17, and 29 are scored in reverse order and the rest in positive order on a 5-point Likert scale. To indicate a concern in the three subscales, the cutoff score of the compassion satisfaction subscale was <37; the cutoff score of the burnout subscale was >27, and the cutoff score of the secondary traumatic subscale was >17. If the score of any one subscale exceeded the cutoff score, it was considered mild compassion fatigue. If the score of any two subscales exceeded the cutoff score, it was categorized as moderate compassion fatigue. If the scores off all three subscales surpassed the cutoff score, it was considered severe compassion fatigue. A total score of this ProQOL was also calculated to indicate the overall level of compassion fatigue. Cronbach’s alpha coefficient for this study was 0.761, which indicated good reliability.

#### 2.5.5. Socio-demographic characteristics

Additional to the aforementioned measures of key variables of interest, socio-demographic data was collected too. A self-developed general information questionnaire was included in the survey to collect nurse socio-demographics, including gender, age, education, marital status, monthly income, job title, years as a clinical nurse, employment type, hospital rank, number of night shifts per week, and daily sleep time.

### 2.6. Statistical analysis

Descriptive statistical analysis was performed to describe the distribution of each variable of interest. Specifically, frequencies and percentages were performed to describe categorical variables, while means and standard deviations were used to describe continuous variables. Pearson correlation coefficients were used to measure the correlation between compassion fatigue, role stress, perceived social support, and emotional regulation efficacy. An analysis of nurses’ compassion fatigue was performed using the independent sample *t*-test or one-way analysis of variance (ANOVA). Parameters outside the limit of ±2 for skewness and ± 7 for kurtosis were considered as an index of non-normality (29). The skewness and kurtosis of the variables in this study were within the above range, which indicated a normal distribution in general. These analyses were carried out with SPSS 25.0.

Finally, we verified and analyzed research hypotheses by constructing structural equation models using AMOS (v23). Fitness of each model was also evaluated using the following indicators: (a) χ^2^/df; (b) Normed fit index (NFI); (c) goodness of fit index (GFI); (d) adjusted goodness of fit index (AGFI); (e) Tucker-Lewis fit index (TLI); (f) comparative fit index (CFI); and (g) root mean square error of approximation (RMSEA). An RMSEA ≤0.08, χ^2^/df < 5, and the rest of the indices, such as NFI ≥ 0.90, GFI ≥ 0.90, AGFI ≥ 0.90, TLI ≥ 0.90 and CFI ≥ 0.90, indicated a satisfactory model fit (41, 42). A *p*-value of <0.05 were considered statistical significance in this study.

## 3. Results

### 3.1. Characteristics of the participants

Forty-two questionnaires were excluded from the 542 submitted questionnaires (a valid rate of 92.25%) mainly due to concerns of data quality and validity. Characteristics of the participants are presented in Table 1. Of the 500 participants, 477 (or 95.4%) were female. Nearly two-thirds (61.6%) were between 26 and 35 years old. The majority of the nurses were with a bachelor’s degree (79.4%), married and with children (59.8%), holding a job title of Level 1 (64.6%), employed under contract (73.2%), and working in tertiary hospitals (64.0%). Nearly half of the nurses (49.4%) had a monthly salary between 3001 and 6000 RMB and worked 1 ~ 3 nightshifts per week (48.6%). Another 18.2% had three or more nightshifts per week. Nearly three-fifths had 5–20 years of working experience (59.0%). Approximately 3 in 5 nurses (61.6%) nurses reported having 4–6 h of sleep per day.

**Table 1:** Sociodemographic information and distribution of compassion fatigue (*n* = 500).

Factors	N (%)	Compassion fatigue M (SD)	*t/F*	*p*
Gender
Male	23 (4.6)	81.65 (11.55)	−0.10	0.92
Female	477 (95.4)	81.88 (10.78)		
Age
≤25	87 (17.4)	84.01 (11.14)	1.99	0.12
26 ~ 35	308 (61.6)	81.18 (10.99)		
36 ~ 45	78 (15.6)	81.47 (9.97)		
≥46	27 (5.4)	84.07 (8.92)		
Educational level
Technical secondary school	7 (1.4)	86.29 (5.56)	3.00	0.03
Junior college	83 (16.6)	80.40 (9.57)		
Undergraduate college	397 (79.4)	81.86 (10.99)		
Postgraduate or above	13 (2.6)	89.31 (11.54)		
Marital status
Unmarried/single	94 (18.8)	81.90 (10.36)	2.70	0.03
Unmarried with a partner	50 (10.0)	85.82 (12.30)		
Married w/kids	299 (59.8)	81.26 (10.54)		
Married w/o kids	45 (9.0)	80.31 (11.50)		
Divorced/Widowed	12 (2.4)	86.33 (7.66)		
Hospital rank
Grade A tertiary hospital	303 (60.6)	81.00 (10.91)	3.16	0.01
Grade B tertiary hospital	17 (3.4)	88.06 (13.35)		
Grade A secondary hospital	146 (29.2)	83.27 (9.70)		
Grade B secondary hospital	16 (3.2)	83.25 (12.47)		
Community hospital	18 (3.6)	78.11 (10.85)		
Job title
Level 1	323 (64.6)	81.51 (11.06)	2.40	0.07
Level 2	153 (30.6)	81.84 (9.84)		
Level 3	20 (4.0)	88.15 (12.56)		
Level 4	4 (0.8)	81.25 (9.88)		
Employment methods
Contract system	366 (73.2)	81.66 (10.90)	1.89	0.15
Personnel agency	69 (13.8)	80.83 (10.26)		
Formal staffing	65 (13.0)	84.18 (10.63)		
Monthly income (RMB)
<3000	40 (8.0)	81.28 (12.09)	0.69	0.56
3001 ~ 6000	247 (49.4)	82.61 (10.41)		
6001 ~ 9000	152 (30.4)	80.93 (11.06)		
9001 ~ 12000	42 (8.4)	81.12 (10.05)		
>12000	19 (3.8)	82.74 (12.67)		
Years of working
<1	23 (4.6)	85.65 (10.17)	2.39	0.07
1 ~ 5	138 (27.6)	82.75 (11.36)		
5 ~ 20	295 (59.0)	80.92 (10.63)		
≥20	44 (8.8)	83.57 (9.87)		
Weekly night shift
0	166 (33.2)	81.26 (10.63)	0.61	0.65
1 ~ 3	243 (48.6)	81.85 (10.67)		
≥3	91 (18.2)	83.05 (11.45)		
Sleep time (hour)
<4	69 (13.8)	85.90 (11.86)	4.30	<0.01
4 ~ 6	308 (61.6)	81.41 (10.21)		
6 ~ 8	109 (21.8)	81.17 (11.43)		
≥8	14 (2.8)	77.79 (8.94)		

### 3.2. Status of nurses’ compassion fatigue, role stress, and psychosocial resources

As shown in Table 2, the average total scores of compassion fatigue, role stress, perceived social support, and emotional regulation efficacy were 81.87 ± 10.80, 36.71 ± 7.44, 62.04 ± 12.77, and 62.79 ± 10.05, respectively. Compassion fatigue was positively correlated with role stress (*r* = 0.62, *p* < 0.01) but negatively related to perceived social support (*r* = −0.33, *p* < 0.01) and emotional regulation efficacy (*r* = −0.40, *p* < 0.01). Role stress was negatively correlated with perceived social support (*r* = −0.30, *p* < 0.01) and emotional regulation efficacy (*r* = −0.39, *p* < 0.01). Perceived social support was positively associated with emotional regulation efficacy (*r* = 0.66, *p* < 0.01).

**Table 2:** Correlation analysis between compassion fatigue, role stress, perceived social support and emotional regulation efficacy of nurses.

Variables	1	2	3	4	Mean (SD)
1. CF	1				81.87 (10.80)
2. RS	0.62**	1			36.71 (7.44)
3. PSS	−0.33**	−0.30**	1		62.04 (12.77)
4. ERE	−0.40**	−0.39**	0.66**	1	62.79 (10.05)

**p < 0.01; CF, compassion fatigue; RS, role stress; PSS, perceived social support; ERE, emotional regulation efficacy.

Mild compassion fatigue was found in 142 nurses (28.4%), moderate compassion fatigue in 139 nurses (27.8%), severe compassion fatigue in 190 nurses (38.0%), and no compassion fatigue in only 29 nurses (5.8%).

### 3.3. The relationship between psychosocial resources, role stress, and compassion fatigue

**Figure 2:**
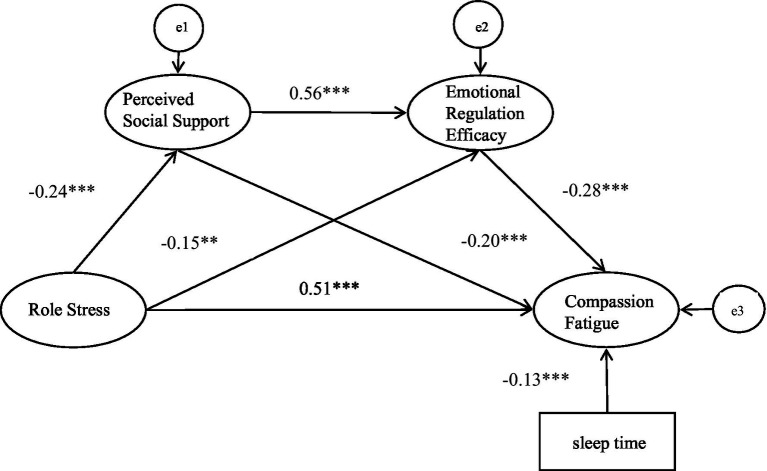
The model of compassion fatigue, emotional regulation efficacy, perceived social support, role stress and demographic factors. ***p < 0.001, **p < 0.01. CMIN/DF = 3.013, NFI = 0.959, GFI = 0.946, AGFI = 0.911, TLI = 0.959, CFI = 0.972, RMSEA = 0.064.

Estimates from the structural equation models are summarized in Table 3. The model fit the data well, as shown in Figure 2. The significance of the potential mediating effects was tested using the bias-corrected Bootstrap method (5000 randomly repeated samples). The significance of the effect was based on the 95% confidence intervals that did not contain zero. The effect was also controlled for socio-demographics of the participants. Before constructing structural equations, the effect of socio-demographics on compassion fatigue was assessed by regression analysis. This result showed that only sleep time (*B* = −2.06, *p* < 0.05) significantly affected compassion fatigue and was included as a control variable in the estimated model (*β* = −0.13, *p* < 0.001).

**Table 3:** Direct, indirect, and total effects of role stress on compassion fatigue.

Pathway	Estimate	95%CI		*p*
		Lower	Upper	
Direct effects				
RS → PSS	−0.24	−0.34	−0.14	***
PSS → ERE	0.56	0.48	0.64	***
ERE → CF	−0.28	−0.37	−0.18	***
RS → ERE	−0.15	−0.25	−0.05	0.002
PSS → CF	−0.20	−0.28	−0.11	***
RS → CF	0.51	0.41	0.60	***
Sleep time→CF	−0.13	−0.20	−0.07	***
Indirect effects				
RS → PSS → CF	0.06	0.03	0.11	***
RS → ERE → CF	0.06	0.02	0.11	0.001
RS → PSS → ERE → CF	0.05	0.03	0.09	***
Total effects				
RS → CF	0.86	0.65	1.09	***

***p < 0.001; RS, role stress; CF, compassion fatigue; PSS, perceived social support; ERE, emotional regulation efficacy.

The result of the model suggested that role stress had a direct effect on compassion fatigue (*β* = 0.51, *p* < 0.001). The indirect influence of role stress on compassion fatigue via its effect on perceived social support and emotional regulation efficacy respectively, was significant (*β* = 0.06, *p* < 0.001; *β* = 0.06, *p* = 0.001). Importantly, the model also indicated a significant chain mediation effect of perceived social support and emotional regulation efficacy (*β* = 0.05, *p* < 0.001).

## 4. Discussion

Using data from a cross-sectional survey, this study examined influencing factors of compassion fatigue among nurses in China during a public health emergency (i.e., the COVID-19 pandemic) and tested the possible mediating roles of perceived social support and emotional regulation efficacy between role stress and compassion fatigue. Our findings provided new insights into the complex relationship between role stress, psychosocial resources (i.e., perceived social support and emotional regulation efficacy), and compassion fatigue. These findings are informative for developing a healthy and competency nursing workforce in China beyond the COVID-19 Pandemic.

Nurses in China experienced unprecedently high compassion fatigue during the COVID-19 pandemic. In our study, more than 9 in 10 nurses reported compassion fatigue, including 65.8% with moderate or severe compassion fatigue. Previously, Hunsaker et al. (43) found that 65.9% of nurses experienced compassion fatigue in a study of 1000 emergency department nurses in the United States. Wang et al. (44) investigated 1123 clinical nurses and found that 88.38% of nurses suffered from compassion fatigue in 2020. Our findings and these results suggest that the occurrence of compassion fatigue is extremely common among nurses. Furthermore, compared to the studies before the COVID-19 pandemic, the rate of compassion fatigue is higher in our study, which suggest that nurses’ compassion fatigue has been exacerbated by the high-intensity, high-risk, high-stress work associated with the COVID-19 pandemic (1). Such a high compassion fatigue is alarming for a healthy and stable nursing workforce as other researchers have indicated that nurses are more likely to leave nursing when they experience compassion fatigue (45). Hospital executives as well as health policymakers should make a commitment to help nurses manage compassion fatigue. Research is also warranted to examine potential long-term consequence of COVID-19 pandemic on the health of frontline nurses, including compassion fatigue and other health issues.

Our study further confirms the positive relationship between role stress and compassion fatigue and provides empirical evidence quantifying this relationship in frontline nurses in China during the public health emergency. Previously, Yates (46) has concluded that job stress results in burnout, one dimension of compassion fatigue. Similarly, Alharbi et al. (17) noted that the COVID-19 outbreak increased nurses’ work stress, which raised the risk of compassion fatigue. The above observations were in line with the results we obtained. Additional to their heavy patient care duties, frontline nurses often have may other roles in life, such as caregivers, parents, husbands or wives, etc., which can add extra stress when they already face tremendous stress working at the frontline battling the pandemic as a direct care provider. Consequently, they are more likely to experience burnout, depression, and anxiety, which are closely tied to compassion fatigue.

The research findings indicate that in addition to the direct impact, role stress also indirectly influences compassion fatigue through perceived social support. According to the stress-buffering theory, high levels of social support can protect individuals from the potential negative effects of stressors (47). This suggests that an increased level of perceived social support is beneficial in alleviating the impact of stress on compassion fatigue. Wu et al. (48) conducted a cross-sectional survey involving 1464 Chinese bank employees and found that perceived social support could mediate the relationship between job stress and burnout. Additionally, through an investigation of 444 Chinese nurses, Liu and Aungsuroch (49) demonstrated that perceived social support acted as a mediator between work stress and burnout. The above results were consistent with the present study. Therefore, in highly stressful clinical environments, hospital managers could implement training activities aimed at enhancing nurses’ perception of social support to reduce their compassion fatigue.

In addition to the mediating role of perceived social support, emotional regulation efficacy also partially mediates the relationship between role stress and compassion fatigue. This finding is in agreement with previous studies. For instance, in a cross-sectional survey of 264 Italian professionals working with refugees, Tessitore et al. (50) found that emotion regulation exerted a mediating effect on the association between secondary traumatic stress and burnout. Similarly, Kshtriya et al. (51) revealed an indirect mechanistic pathway suggesting that the mediator, an emotion regulation strategy that expressed inhibition, increased the negative impact of occupational stress on the psychological well-being of emergency personnel when it was enhanced. The current research provides support for the mediating role of emotional regulation efficacy, which may be explained using a person-by-situation approach to the emotion regulation model. According to this model, individual reconstruction of emotions can lead to better mental health outcomes when exposed to high-stress environments that are relatively uncontrollable (52). This suggests that hospital administrators need to provide education and training for nurses to enhance the regulation of emotional abilities and alleviate potential adverse psychological health outcomes.

This research offered new understanding that the chain mediating effect of perceived social support and emotional regulation efficacy on the relationship between role stress and compassion fatigue. In this study, nurses were exposed to high-pressure clinical environments due to their heavy workloads, caring for critically ill patients, and performing duties in multiple roles. However, perceived social support and emotional regulation efficacy are positive psychosocial qualities acting as internal protective factors for individuals (22, 29). Researchers believed that individuals with a higher perception of social support can typically regulate their emotions better (53), which may reduce the effects of stress on compassion fatigue. When individuals face stress in their personal or professional lives, positive psychosocial resources may enhance their psychosocial resilience (53, 54). This resilience empowers individuals with courage and perseverance to confront negative thoughts generated by these stressors (13, 55). Therefore, strengthening positive psychosocial resources is crucial for maintaining psychological well-being. This pathway, which we have found, precisely explains the effectiveness of these psychological interventions during the COVID-19 pandemic. This also points to the need to deeply investigate the mechanisms and scientifically set up interventions. In the future, we need to dig deeper into positive psychosocial resources to broaden our understanding of the mechanism of compassion fatigue.

Additional to the aforementioned factors, our study also found sleep time was an important factor significantly associated with compassion fatigue. Similar to Stewart et al. (56), compassion fatigue could occur when nurses did not get enough sleep. Studies have shown that sleep deficit may cause a range of physiological stress responses that alter the composition of the gut microbiota. The altered microbiota may affect the function of the nervous and immune systems, thereby reducing an individual’s ability to cope with psychological and physical stress and making him or her more susceptible to stressful life events (57). It suggests that managers should arrange shifts scientifically and mobilize human resources reasonably, which is beneficial for nurses’ physical and mental health.

Findings from this study have several implications for nursing management, research, and policies. According to our results, perceived social support and emotional regulation efficacy have a positive protective effect against compassion fatigue. Therefore, nursing managers need to strengthen nurses’ construction of social support comprehension and enhance their emotional regulation abilities to alleviate compassion fatigue. On one hand, nursing managers could enhance nurses’ perception of harmonious interpersonal environments through some methods such as mindfulness interventions (58) and peer support programs (59). The enhancement of this comprehension of social support in interpersonal interactions helps shift nurses’ negative perceptions of stress to a positive focus on stress, thereby improving nurses’ well-being. On the other hand, nursing managers could enhance nurses’ emotional regulation abilities through activities like Yoga (60) and Balint group exercises (61), which include expressing positive feelings and managing negative emotions. Simultaneously, nurses can seek social support from family, friends, and organizations to maintain stable emotions and expand positive psychosocial resources.

## 5. Strengths and limitations

This study enriched positive psychology and deeply explored the mechanism of compassion fatigue from the perspective of positive psychosocial resources. It turns out that perceived social support and emotional regulation efficacy play an essential role in buffering compassion fatigue, which provides a meaningful reference for psychological intervention for nurses in the future.

Despite the aforementioned strengths, this study has several limitations that should be noted. First, this study is a cross-sectional survey study, therefore our findings are limited to correlation relationship rather than causal relationships. It thus is necessary to conduct longitudinal studies in the future to explore the causal relationship between role stress, psychosocial resources, and compassion fatigue. Second, we only surveyed nurses in hospitals in Hubei Province. Given the geographic diversity in China, our findings may not be generalizable to nurses in other parts of China. Large-scale at national level should be considered in the future.

## 6. Conclusion

Nurses experience high level of compassion fatigue, a condition that is widespread and requires more attention. This study used structural equation modeling and found that when nurses are in high role stress circumstances, perceived social support and emotional regulation efficacy may buffer the negative effects of role stress on compassion fatigue. Moreover, it indicates that by building a excellent support system and managing emotions effectively, people can enhance their psychological well-being and activate positive forces within themselves. This will help nurses deal with role stress associated with the outbreak and improve compassion fatigue. It also helps to provide thoughts on promoting the mental health of nurses when uncertain public health event occurs in the future.

## Data availability statement

The datasets presented in this article are not readily available. However, the data that support the findings of this study are available from the corresponding author upon reasonable request. Requests to access the datasets should be directed to HH, hhjiabei@hbtcm.edu.cn.

## Ethics statement

The studies involving humans were approved by Hubei University of Chinese Medicine (Approval: HBZY2020-C14-01).

## Author contributions

YZ: Formal analysis, Writing – original draft, Writing – review & editing. HH: Conceptualization, Methodology, Writing – review & editing. CY: Writing – review & editing. XW: Investigation, Writing – review & editing, Data curation. JL: Formal analysis, Validation, Writing – original draft. JX: Data curation, Investigation, Writing – review & editing. BF: Data curation, Investigation, Writing – review & editing. YC: Data curation, Formal analysis, Writing – original draft. CM: Conceptualization, Writing – review & editing.
