# Corrigendum: Chain mediations of perceived social support and emotional regulation efficacy between role stress and compassion fatigue: insights from the COVID-19 pandemic

**DOI:** 10.3389/fpubh.2023.1346956

**Published:** 2024-01-04

**Authors:** Yuan Zhang, Huijuan He, Chongming Yang, Xiangrong Wang, Jiang'an Luo, Jie Xiao, Bei Fu, Yiwen Chen, Chenjuan Ma

**Affiliations:** ^1^School of Nursing, Hubei University of Chinese Medicine, Wuhan, Hubei, China; ^2^College of Family, Home, and Social Sciences, Brigham Young University, Provo, UT, United States; ^3^NYU Rory Meyers College of Nursing, New York, NY, United States

**Keywords:** COVID-19, compassion fatigue, emotional regulation efficacy, nurses, perceived social support, role stress

In the published article, there was an error in Table 1. Instead of “tertiary hospital, secondary hospital and community hospital”, it should be “Grade A tertiary hospital, Grade B tertiary hospital, Grade A secondary hospital, Grade B secondary hospital and community hospital.” The corrected Table 1 appears below.

**Table 1:** Sociodemographic information and distribution of compassion fatigue (*n* = 500).

**Factors**	**N(%)**	**Compassion fatigue M(SD)**	***t/F***	***p***
**Gender**				
Male	23 (4.6)	81.65 (11.55)	−0.10	0.92
Female	477 (95.4)	81.88 (10.78)		
**Age**				
≤ 25	87 (17.4)	84.01 (11.14)	1.99	0.12
26–35	308 (61.6)	81.18 (10.99)		
36–45	78 (15.6)	81.47 (9.97)		
≥46	27 (5.4)	84.07 (8.92)		
**Educational level**				
Technical secondary school	7 (1.4)	86.29 (5.56)	3.00	0.03
Junior college	83 (16.6)	80.40 (9.57)		
Undergraduate college	397 (79.4)	81.86 (10.99)		
Postgraduate or above	13 (2.6)	89.31 (11.54)		
**Marital status**				
Unmarried/single	94 (18.8)	81.90 (10.36)	2.70	0.03
Unmarried with a partner	50 (10.0)	85.82 (12.30)		
Married w/kids	299 (59.8)	81.26 (10.54)		
Married w/o kids	45 (9.0)	80.31 (11.50)		
Divorced/Widowed	12 (2.4)	86.33 (7.66)		
**Hospital rank**				
Grade A tertiary hospital	303 (60.6)	81.00 (10.91)	3.16	0.01
Grade B tertiary hospital	17 (3.4)	88.06 (13.35)		
Grade A secondary hospital	146 (29.2)	83.27 (9.70)		
Grade B secondary hospital	16 (3.2)	83.25 (12.47)		
Community hospital	18 (3.6)	78.11 (10.85)		
**Job title**				
Level 1	323 (64.6)	81.51 (11.06)	2.40	0.07
Level 2	153 (30.6)	81.84 (9.84)		
Level 3	20 (4.0)	88.15 (12.56)		
Level 4	4 (0.8)	81.25 (9.88)		
**Employment methods**				
Contract system	366 (73.2)	81.66 (10.90)	1.89	0.15
Personnel agency	69 (13.8)	80.83 (10.26)		
Formal staffing	65 (13.0)	84.18 (10.63)		
**Monthly income (RMB)**				
< 3,000	40 (8.0)	81.28 (12.09)	0.69	0.56
3,001–6,000	247 (49.4)	82.61 (10.41)		
6,001–9,000	152 (30.4)	80.93 (11.06)		
9,001–12,000	42 (8.4)	81.12 (10.05)		
>12,000	19 (3.8)	82.74 (12.67)		
**Years of working**				
< 1	23 (4.6)	85.65 (10.17)	2.39	0.07
1–5	138 (27.6)	82.75 (11.36)		
5–20	295 (59.0)	80.92 (10.63)		
≥20	44 (8.8)	83.57 (9.87)		
**Weekly night shift**				
0	166 (33.2)	81.26 (10.63)	0.61	0.65
1–3	243 (48.6)	81.85 (10.67)		
≥3	91 (18.2)	83.05 (11.45)		
**Sleep time (hour)**				
< 4	69 (13.8)	85.90 (11.86)	4.30	< 0.01
4–6	308 (61.6)	81.41 (10.21)		
6–8	109 (21.8)	81.17 (11.43)		
≥8	14 (2.8)	77.79 (8.94)		

The authors apologize for this error and state that this does not change the scientific conclusions of the article in any way. The original article has been updated.

